# Partial trisomy 16q and partial monosomy 7p of a fetus derivated from paternal balanced translocation: A case report

**DOI:** 10.1097/MD.0000000000024382

**Published:** 2021-02-19

**Authors:** Hui-Hui Xie, Tong Liu, Jing-Bo Zhang, Jing-Fang Zhai, Ying Liu

**Affiliations:** Department of Prenatal Diagnosis Medical Center of Xuzhou Central Hospital, Xuzhou Clinical Schools of Xuzhou Medical University and Nanjing Medical University, Xuzhou, Jiangsu, China.

**Keywords:** copy number variation sequencing, karyotyping, noninvasive preliminary screening, partial monosomy 7p, partial trisomy 16q

## Abstract

**Introduction:**

Subchromosomal deletions and duplications could currently be detected by noninvasive preliminary screening (NIPS). However, NIPS is a screening test that requires further diagnosis. Here we report a fetus with an autosomal abnormality revealed by NIPS and conventional karyotype combined with copy number variations sequencing (CNV-seq) confirmed the fetus with an unbalanced translocation.

**Patient concern:**

This was the fourth pregnancy of a 30-year-old woman who underwent 2 spontaneous abortions and gave birth to a child with a normal phenotype. The woman and her husband were healthy and nonconsanguineous. NIPS indicated a repeat of about 19-Mb fragment at the region of 16q22.1-q22.4 at 17-week gestation.

**Diagnoses:**

The combination of traditional karyotype and CNV-seq could better locate the abnormal chromosomal region and further identify the source of fetal chromosomal abnormalities. Simultaneously, we evaluated the fetal morphology by ultrasound examination. The karyotype of the fetus was 46,XX,der(7)t(7;16)(p22;q23) and CNV-seq results showed an approximately 20.96-Mb duplication in 16q22.1-q24.3 (69200001-90160000) and an approximately 3.86-Mb deletion in 7p22.3-p22.2 (40001-3900000). Prenatal ultrasound revealed the fetal micrognathia. The paternal karyotype was 46,XY, t (7;16) (p22;q23), while the maternal was normal. The fetus inherited an abnormal chromosome 7 from its father.

**Interventions:**

No treatment for the fetus.

**Outcomes:**

Pregnancy was terminated.

**Conclusions:**

To our knowledge, the occurrence of de novo partial trisomy 16q (16q22.1-qter) and partial monosomy 7p (7p22.2-pter) has not previously been reported up to now. Here, we present the perinatal findings of such a case and a review of the literatures. CNV-seq combined with karyotype is a useful tool for chromosomal abnormalities indicated by NIPS.

## 1 Introduction

Noninvasive preliminary screening (NIPS), also known as noninvasive prenatal screening, is based on the analysis of cell-free fetal DNA in maternal blood. Since its introduction in 2011, NIPS for fetal aneuploidy has rapidly become a first-level screening test in clinical practice. The main advantages of NIPS are its good sensitivity and specificity to trisomy 21, 13, and 18.^[1]^ In addition, the results of NIPS based on low-depth whole-genome sequencing can also be used to indicate other chromosomal abnormalities including aneuploidy of other chromosomes and copy number variations (CNVs). However, due to low production of cell-free fetal DNA from placental origin or low sequencing depth, NIPS yields false negative and false positive results in the detection of CNVs.^[2]^ Therefore, further diagnostic means is needed. CNV-seq is a high-resolution whole-genome screening technology that can be used to analyze the presence of CNVs. In this study, we report a fetus with a 19-Mb duplication on chromosome 16 indicated by NIPS, who was conformed to be a paternal balanced translocator through amniocentesis karyotype, CNV-seq, and the genetic analysis of proband family members. Meanwhile, we analyze the ultrasonic abnormal phenotype possibly related to the result of CNV-seq in molecular biology genetics.

## 2 Case presentation

**Figure 1:**
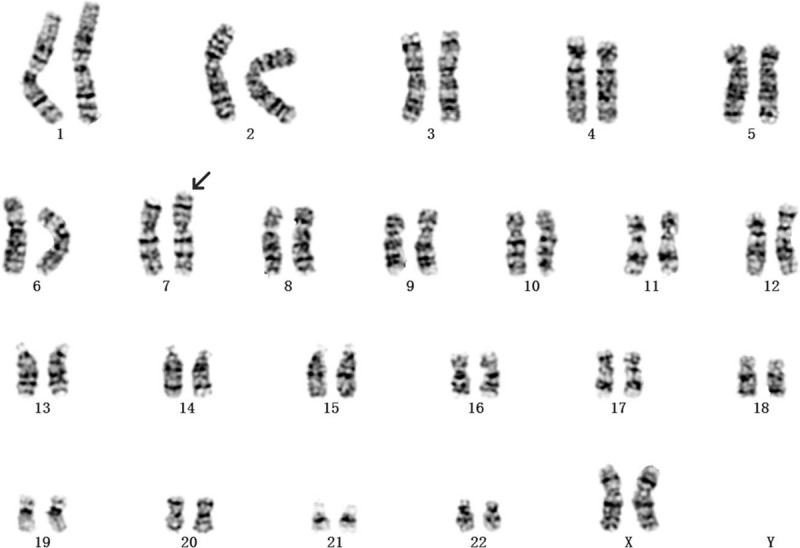
The fetal karyotype was 46,XX,der(7)t(7;16)(p22;q23) at the level of 300 to 400 bands. The arrows indicate the breakpoints.

**Figure 2:**
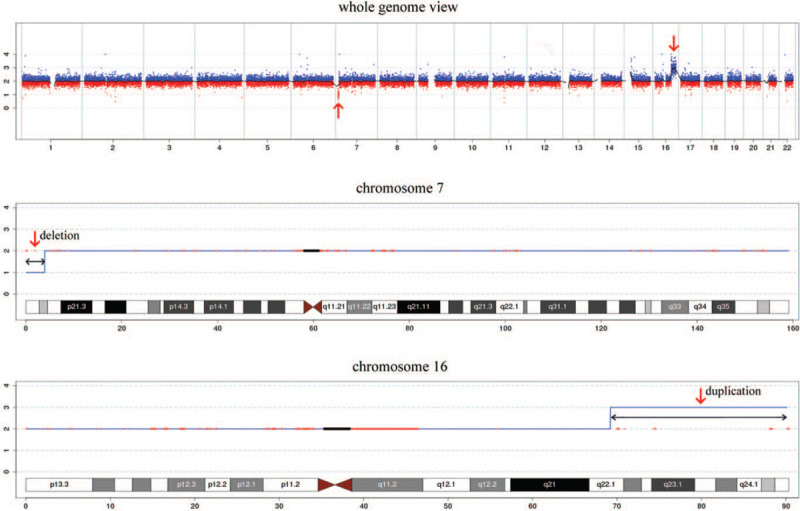
The fetal results of CNV-seq showed a 3.86-Mb deletion (40001-3900000) in 7p22.3-p22.2 and a 20.96-Mb duplication (69200001-90160000) on the chromosome in 16q22.1-q24.3. The arrows indicate the breakpoints. CNV-seq = copy number variations sequencing.

**Figure 3:**
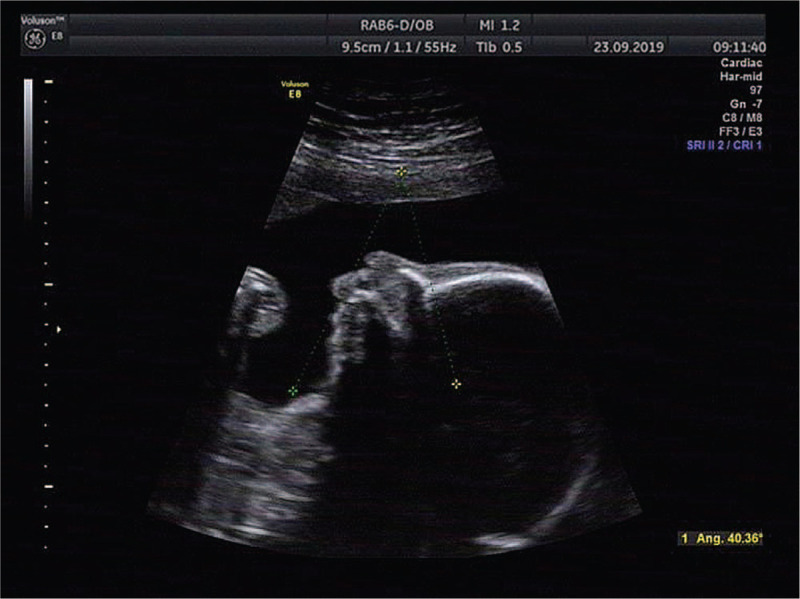
The fetal ultrasound imaging showed micrognathia.

**Figure 4:**
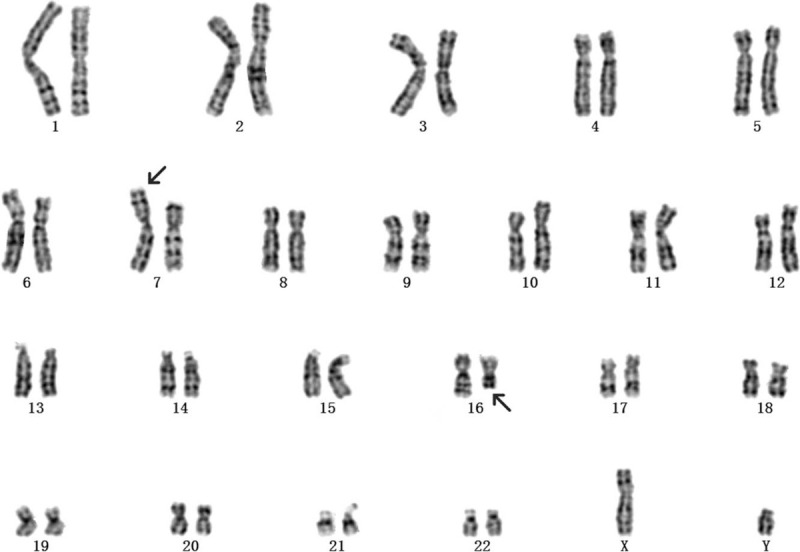
The father's peripheral blood karyotype was 46,XY,t(7;16)(p22;q23) at the level of 300 to 400 bands. The arrows indicate the breakpoints.

This was the fourth pregnancy of a 30-year-old gravida 4, para 1, healthy woman who had experienced 2 spontaneous abortions and delivered a phenotypically normal child. The woman and her husband were healthy and nonconsanguineous. When the second missed abortion occurred, the doctor recommended that the couple have the peripheral blood karyotype test. However, the couple refused because they had decided not to have children any more. However, an unplanned pregnancy happened. NIPS indicated a repeat of about 19-Mb fragment at the region of 16q22.1-q22.4 at 17-week gestation. After consultation, she opted for prenatal karyotyping by amniocentesis. Amniotic cells cultured by G-banding showed an additional unidentified substance on chromosome 7 (Fig. 1). The International System for Human Cytogenetic Nomenclature 2016 nomenclature was used to describe karyotypes. Simultaneously, the CNV-seq results of uncultured amniotic cells showed that a 3.86-Mb deletion (40001-3900000) in 7p22.3-p22.2 and a 20.96-Mb duplication (69200001-90160000) in 16q22.1-q24.3 (Fig. 2). Prenatal ultrasound at 21-week gestation revealed the fetal structural abnormalities presented with micrognathia (Fig. 3). The mother and sister had normal karyotype analysis, while the father's karyotype was 46,XY,t(7;16)(p22;q23) (Fig. 4). Taken together, these molecular cytogenetic experiments enabled us to determine the presence of an unbalanced translocation in the fetus, 46,XX,der(7)t(7;16)(p22;q23). A balanced translocation of the father's chromosomes leads to chromosomal abnormalities in the fetus. We informed the parents about the possible consequences of chromosomal abnormalities. After consultation, the parents chose to terminate the pregnancy. The autopsy was rejected by the parents. Patient has provided informed consent for publication of the case. This study was approved by the ethics committee of Xuzhou central hospital.

## 3 Discussion

More recently, some laboratories have expanded NIPS to detect CNVs.^[2,3]^ However, false positives caused by the influence of mother, fetus or placenta simultaneously exist due to limitations of the technology. In a retrospective study of 8152 pregnant women, they analyzed the subchromosomal microdeletions and microduplications, and only 13 (36.11%) of the 51 positive cases (0.63%) were true positive.^[3]^ Therefore, all abnormal NIPS results are required further prenatal diagnosis. Karyotype analysis is a classical, commonly used approach in prenatal diagnosis, and specific chromosomal structures such as balanced reciprocal translocations can be identified at relatively low cost. However, due to the low resolution, this method has obvious limitations in detecting CNVs. In contrast, CNV-seq has a higher resolution and can effectively detect chromosomal microdeletions and microduplications. However, CNV-seq fails to discover low levels of chromosomal mosaicism and balanced reciprocal translocations. Here, the fetus with 16q duplication and 7p deletion due to paternal balanced translocation was detected by traditional karyotyping combined with CNV-seq. The occurrence of concurrent partial trisomy 16q (16q22.1-qter) and partial monosomy 7p (7p22.2-pter) has not previously been reported up to now.

In most cases, trisomy 16q is due to a malsegregation of maternal balanced translocation and rarely to paternal balanced translocation.^[4–6]^ Prenatal diagnosis of trisomy 16 or trisomy 16q is uncommon.^[7]^ Since the first case was reported, 47 patients with partial trisomy 16q or trisomy 16q have been reported. We reviewed 47 cases and summarized that the most common malformations of partial trisomy 16q were low birth weight, hypotonia, failure to thrive, high/prominent forehead, broad/depressed nasal bridge, low set/dysplastic ears, micrognathia, foot deformity, congenital heart disease and mental/psychomotor retardation (Table 1).^[8–40]^ To our knowledge, only 8 cases of partial trisomy 16q (16q22-qter) have been reported in the literature.^[27–34]^ The clinical picture of the distal duplication of 16q tends to be closer to that of complete duplication(16q) and to be severer than the proximal duplication, since the heterochromatic region 16q11-q12 has no effect on the phenotype.^[26]^ Houlston RS described an infant with a de novo karyotype of 46,XX,der(15)t(15;16)(q26.1;q22) had multiple malformations such as intrauterine growth retardation, dislocation of the left knee and hip, stiffness of the elbow joints, choanal atresia, high forehead, narrow palpebral fissures, antimongoloid slant, midface hypoplasia, micrognathia, high-arched palate, anal stenosis, blind ending sinus in the coccygeal area, small 5th fingers, delayed motor development.^[31]^ In our case, the fetal ultrasound also showed microjaw deformity. Therefore, we speculated that micrognathia might be caused by partial trisomy 16q. In the present case, the region of 16q22.1–16q24.3 contains >150 Online Mendelian Inheritance in Man (OMIM) genes including 57 morbidity-associated genes (Table 2) (https://decipher.sanger.ac.uk/browser). Chromatin licensing and DNA replication factor 1 (CDT1) (OMIM: 605525), located at 16q24.3, is required for DNA replication at multiple stages of development and mitosis.^[41]^ CDT1 has been indicated to be associated with Meier-Gorlin syndrome 4.^[42]^ Patients with this syndrome usually have distinctive facial features, full lips, low-set/rotated ears, micrognathia, short stature, patellar aplasia/hypoplasia, narrow nose with a high nasal bridge and abnormalities in sexual development. So we hypothesized that this phenotype might be related to CDT1. In addition, ankyrin repeat domain 11 (OMIM: 611192) at 16q24.3 has been shown to be associated with KBG syndrome.^[43]^ Patients typically have facial deformities, upper and middle incisors, skeletal (mainly costal) deformities, and developmental delays. The gene may also be involved in the phenotype of partial trisomy 16q.

**Table 1:** Comparison of clinical features of 47 patients with complete or partial trisomy 16q reported in the literature.

16q trisomy	q11→qter	q12→qter	q13→qter	q21→qter	q22→qter	q23→qter	q24→qter
	N = 12	N = 4	N = 5	N = 6	N = 8	N = 8	N = 4
Reference	8–16	17–20	21–24	25–30	31–38	28, 39–43	28, 44, 45
Low birth weight	7	2	5	5	6	6	1
Survival	12 d→1.5 yr^∗^	10 d→1.5 mo	8 d→2 yr^∗^	10 mo→3.5 yr^∗^	7 mo→10 yr^∗^	3 mo→15 yr^∗^	3 yr^∗^→19 yr^∗^
Hypotonia	3		2	5	3	5	1
Failure to thrive	6		2	2	5	4	2
Periorbital edema	3			1			1
Abnormal skin	4		1	1	2		
Bitemporal narrowing	2	1			1		1
High/prominent forehead	7	4	2	5	7	4	2
Hypertelorism	3	1		2	1	1	
Downslanting palpebral fissures	8		1	2	2	1	1
Small palpebral fissures	4	1		3	2	1	1
Broad/depressed nasal bridge	2	2	1	3	3	6	1
Beaked nose	1	1	1	2			1
Low set/dysplastic ears	9	4	5	4	5	7	3
Long philtrum	2		2	2			1
High arched palate	3	1	2	1	3	1	1
Cleft palate	1		2	1	2		
Thin upper lip	4	3	1		5	7	
Epicanthal folds			1	2	4	2	2
Micrognathia	7	4	4	3	5	2	1
Clinodactyly of 5th fingers	5	2	1	1	3		
Short middle phalanx of 5th fingers	3		1				1
Flexion of fingers	5		2	1	3	1	
Flexion /contractures of joints	6		1	1	2	1	
Foot deformity	7	1	3	2	3	7	
Short neck	3	2	1	2	5	1	1
Genital hypoplasia	7	1	1	4	3	3	1
Anus anteposition/imperforate	3	1	1	2	2		1
Congenital heart disease	8	1	5	3	3	4	1
Gut anomalies: short gut/malrotation	3		1			1	1
Vertebral/anomalies	3		1	1	3	1	1
Renal anomalies	4	1	2		4	1	1
Brain anomalies	4			1		1	
Lung anomalies	2		1				
Liver anomalies	2		1				
Gall bladder agenesis	2		1	1			
Abnormal feeding	4		2	5			1
Mental/psychomotor retardation	4		2	4	7	5	3
Agenesis of corpus callosum					1		1
Telecanthus					1		

**Table 2 Genes in the region of 16q22.1-16q24.3 and the associated diseases.** 

Gene	Location	OMIM	Phenotype
*COG8*	16q22.1	606979	Congenital disorder of glycosylation; type IIh
*NQO1*	16q22.1	125860	Leukemia; susceptibility to Breast cancer; poor survival after chemotherapy; Benzene toxicity
*MIR140*	16q22.1	611894	Spondyloepiphyseal dysplasia, Nishimura type
*AARS1*	16q22.1	601065	Charcot-Marie-Tooth disease; Epileptic encephalopathy
*FCSK*	16q22.1	608675	Congenital disorder of glycosylation with defective fucosylation 2
*COG4*	16q22.1	606976	Saul-Wilson syndrom; Congenital disorder of glycosylation, type Iij
*VAC14*	16q22.1-q22.2	604632	Striatonigral degeneration
*HYDIN*	16q22.2	610812	Ciliary dyskinesia
*TAT*	16q22.2	613018	Tyrosinemia, type II
*DHODH*	16q22.2	126064	Miller syndrome
*HP*	16q22.2	140100	Hypohaptoglobinemia; Anhaptoglobinemia
*DHX38*	16q22.2	605584	Retinitis pigmentosa 84
*PMFBP1*	16q22.2	618085	Spermatogenic failure 31
*ZFHX3*	16q22.2-q22.3	104155	Prostate cancer, somatic
*KTCN2*	16q22.1-q22.3	608932	Keratoconus 2
*RFWD3*	16q23.1	614151	Fanconi anemia
*FA2H*	16q23.1	611026	Spastic paraplegia
*LDHD*	16q23.1	607490	D-lactic aciduria
*TMEM231*	16q23.1	614949	Joubert syndrome; Meckel syndrome
*KARS1*	16q23.1	601421	Deafness; Charcot-Marie-Tooth disease
*ADAMTS18*	16q23.1	607512	Microcornea, myopic chorioretinal atrophy, and telecanthus
*WWOX*	16q23.1-q23.2	605131	Epileptic encephalopathy; Spinocerebellar ataxia; Esophageal squamous cell carcinoma
*MAF*	16q23.2	177075	Cataract; Ayme-Gripp syndrome
*GCSH*	16q23.2	238330	Glycine encephalopathy
*BCO1*	16q23.2	605748	Hypercarotenemia and vitamin A deficiency
*GAN*	16q23.2	605379	Giant axonal neuropathy
*PLCG2*	16q23.3	600220	Familial cold autoinflammatory syndrome; Autoinflammation, antibody deficiency; immune dysregulation syndrome
*MLYCD*	16q23.3	606761	Malonyl-CoA decarboxylase deficiency
*SLC38A8*	16q23.3	615585	Foveal hypoplasia 2, with or without optic nerve misrouting and/or anterior segment dysgenesis
*MBTPS1*	16q23.3-q24.1	603355	Spondyloepiphyseal dysplasia
*DNAAF1*	16q24.1	613190	Ciliary dyskinesia
*IRF8*	16q24.1	601565	Immunodeficiency 32A/Immunodeficiency 32B
*FOXF1*	16q24.1	601089	Alveolar capillary dysplasia with misalignment of pulmonary veins
*FBXO31*	16q24.2	609102	Mental retardation
*JPH3*	16q24.2	605268	Huntington disease
*CA5A*	16q24.2	114761	Hyperammonemia due to carbonic anhydrase VA deficiency
*ZNF469*	16q24.2	612078	Brittle cornea syndrome
*CYBA*	16q24.2	608508	Chronic granulomatous disease, autosomal
*MVD*	16q24.2	603236	Porokeratosis
*IHPS5*	16q24.3	612525	Pyloric stenosis; infantile hypertrophic
*CTU2*	16q24.3	617057	Microcephaly, facial dysmorphism, renal agenesis, and ambiguous genitalia syndrome
*PIEZO1*	16q24.3	611184	Lymphatic malformation
*CDT1*	16q24.3	605525	Meier-Gorlin syndrome 4
*APRT*	16q24.3	102600	Adenine phosphoribosyltransferase deficiency
*GALNS*	16q24.3	612222	Mucopolysaccharidosis
*TRAPPC2L*	16q24.3	610970	Encephalopathy, episodic rhabdomyolysis
*ACSF3*	16q24.3	614245	Combined malonic and methylmalonic aciduria
*CDH15*	16q24.3	114019	Mental retardation
*ANKRD11*	16q24.3	611192	KBG syndrome
*PGN*	16q24.3	602783	Spastic paraplegia
*RPL13*	16q24.3	113703	Spondyloepimetaphyseal dysplasia
*CHMP1A*	16q24.3	164010	Pontocerebellar hypoplasia
*CDK10*	16q24.3	603464	Al Kaissi syndrome
*FANCA*	16q24.3	607139	Fanconi anemia
*MC1R*	16q24.3	155555	Skin/hair/eye pigmentation
*TUBB3*	16q24.3	602661	Fibrosis of extraocular muscles; Cortical dysplasia
*GAS8*	16q24.3	605178	Ciliary dyskinesia

In addition to partial chromosome 16 duplication, the fetus also had a 3.86-Mb deletion in 7p22.3p22.2. Andrea C. Yu reported 5 patients with microdeletions at 7p22.3p22.2.^[44]^ The most facial features in these patients include a broad nasal root, a prominent forehead a prominent glabella and arched eyebrows, micrognathia, metopic ridging or craniosynostosis, cleft palate, cardiac defects, and mild hypotonia. Micrognathia is also present, but no gene is found to be associated with this phenotype in this region. The region of 7p22.2-pter contains about 30 OMIM genes including 11 morbidity-associated genes (Table 3). Only eukaryotic translation initiation factors 3B (OMIM: 603917) has been shown to be related with head abnormalities and heart defects after mutations in zebrafish. It is a complex that plays an important role in initiation of translation.^[45]^ However, the role of eukaryotic translation initiation factors 3B in human development is not yet known. Other genes that may be associated with the 7p22.3p22.2 microdeletion syndrome phenotype are the following. Mutations in breast cancer 1-associated ataxia telangiectasia mutated (ATM) activator 1(OMIM: 614506), located in 7p22.3, may have a key role in the protein stability of ATM, which affects neural stem cell differentiation. Breast cancer 1-associated ATM activator 1have been indicated to be associated a severe phenotype known as rigidity and multifocal seizure syndrome, characterized by intractable seizures, hypertonia, autonomic instability, and early death.^[46]^ Mutations in integrator complex subunit 1(OMIM: 611345), located at 7p22.3, have been indicated to be associated the distinctive phenotype as manifested absent or severely limited speech, an abnormal gait, hypotonia, and cataracts.^[47]^ Whether haploinsufficiency of any *OMIM* gene necessarily leads to clinical phenotype in humans requires us to further summarize and track more cases. In this case, the fetus had trisomy 16q and monomer 7p. The genetic abnormalities of these 2 chromosome alterations may interact to making the individual phenotypic consequences. Through data collection, we can indirectly infer possible phenotypes of the fetus. With the exception of the micromaxillary deformity suggested by ultrasound, the detailed features and mental state were not appreciated because the woman's parents eventually terminated the pregnancy and refused autopsy.

**Table 3:** Genes in the region of 7p22.2-7p22.3 and the associated diseases.

*Gene*	Location	OMIM	Phenotype
*CPVT3*	7p22-p14	614021	Ventricular tachycardia
*HYPT10*	7p22.3-p21.3	614238	Hypotrichosis
*FAM20C*	7p22.3	611061	Raine syndrome
*DNAAF5*	7p22.3	614864	Ciliary dyskinesia
*INTS1*	7p22.3	611345	Neurodevelopmental disorder
*MAD1L1*	7p22.3	602686	Prostate cancer, Lymphoma
*MRM2*	7p22.3	606906	Mitochondrial DNA depletion syndrome
*LFNG*	7p22.3	602576	Spondylocostal dysostosis
*BRAT1*	7p22.3	614506	Neurodevelopmental disorder
*IQCE*	7p22.3	617631	Polydactyly
*CARD11*	7p22.2	607210	Immunodeficiency

## 4 Conclusion

In conclusion, precise definitions of subtle chromosome variations should be detected by genome-wide diagnosis such including CNV-seq or chromosome microarray analysis. Meanwhile, ultrasonography can evaluate the malformations of fetus in time. In this report, CNV-seq combined with amniocentesis karyotype is a useful tool to discover the genomic imbalance indicated by NIPS. Through literature analysis, we also analyzed the phenotypes that may be caused by partial trisomy 16q and partial monosomy 7p. The reasonable prenatal screening and prenatal diagnosis can discover some serious birth defects in time, provide accurate prenatal consultation for pregnant women, guide the next pregnancy, and so as to effectively improve the quality of the birth population.

## Author contributions

**Conceptualization:** Huihui Xie, Tong Liu.

**Formal analysis:** Huihui Xie.

**Funding acquisition:** Jing-Fang Zhai.

**Project administration:** Jing-Bo Zhang, Jing-Fang Zhai.

**Supervision:** Jing-Fang Zhai.

**Validation:** Jing-Fang Zhai.

**Visualization:** Huihui Xie, Tong Liu, Ying Liu.

**Writing – original draft:** Huihui Xie.
